# Non-suicidal Self-Injury in Adolescence

**DOI:** 10.1007/s11920-017-0767-9

**Published:** 2017-03-17

**Authors:** Rebecca C. Brown, Paul L. Plener

**Affiliations:** Department of Child and Adolescent Psychiatry and Psychotherapy, University of Ulm, Steinhoevelstr. 5, 89075 Ulm, Germany

**Keywords:** Non-suicidal self-injury, NSSI, Self-harm, Adolescents, Suicidality

## Abstract

**Purpose of Review:**

Non-suicidal self-injury (NSSI) is a common mental health threat among adolescents. This review aims to present the current literature on epidemiology, etiology, and therapeutic approaches with a focus on the period of adolescence.

**Recent Findings:**

NSSI is widespread among adolescents both in community as well as in clinical settings with lifetime prevalence rates between 17 and 60% in recent studies. It is influenced by multiple factors including social contagion, interpersonal stressors, neurobiological background, as well as emotional dysregulation and adverse experiences in childhood.

**Summary:**

There is still a lack of studies regarding the psychotherapeutic as well as the psychopharmacological treatment of NSSI in adolescence. Furthermore, sufficient evidence for prevention programs is missing.

## Introduction

Non-suicidal self-injury (NSSI) is defined as the intentional, self-inflicted damage to the surface of the body without suicidal intent, which is not socially sanctioned [1]. This definition excludes accidental and indirect self-injurious behaviors (e.g., disordered eating or drug abuse), suicidal behaviors, as well as socially accepted behaviors like tattooing, piercing, or religious rituals. The most common methods endorsed in are cutting, scratching, hitting or banging, carving, and scraping [2]. NSSI is particularly present in mid-adolescence [3] and therefore presents a major concern for child and adolescent psychiatrists and psychotherapists, youth welfare workers, teachers, and other professionals working with adolescents, as well as for families of affected youth. In recent years, the body of research on NSSI has rapidly grown. The following review provides an overview of current literature concerning the epidemiology, etiology, risk-factors, and functions of NSSI. It also presents recent findings and recommendations for the treatment of NSSI in adolescence. In the last chapter of this review, the ongoing discussion of including NSSI as an independent disorder in the DSM-5 [4] is reflected on.

## Epidemiology

The first study on the prevalence of NSSI in a school sample of adolescents was published in 2002 [5], which found a prevalence of “self-mutilation” of around 14%. Although there is only little knowledge about the prevalence of NSSI before the early 2000s, prevalence rates have been rather stable across publications from different countries within the past 15 years [6••] when controlling for methodological differences. Methodological differences associated with differences in prevalence rates were for example assessment tools, definition of NSSI, and incentives for participation. International lifetime prevalence rates in adolescents of 17–18% for at least one incidence of NSSI were found in two independent systematic reviews [6••, 7]. Rates of adolescents meeting DSM-5 criteria (see below) are lower and range around 1.5 to 6.7% in child and adolescent community samples [2]. In adolescent psychiatric samples, prevalence rates of NSSI have found to be as high as 60% for one incident of NSSI and around 50% for repetitive NSSI [8]. NSSI not only can occur in the broad context of psychiatric disorders (affective disorders, BPD, substance abuse, anxiety disorder, posttraumatic stress disorders) but can also occur without comorbid psychiatric diagnosis [9–11]. To date, only few studies on the longitudinal development of NSSI exist. One Australian longitudinal study was able to show that self-harming behaviors (including suicidal behaviors) significantly decrease from adolescence (around 15 years of age) to young adulthood (around 29 years of age) [12•]. A systematic review on longitudinal studies on NSSI showed that prevalence rates of NSSI peak around mid-adolescence (around 15–16 years) and decline towards late adolescence (around 18 years) [3].

Although NSSI decreases significantly in late adolescence, adolescents with repetitive NSSI seem to be at high risk to be continuing dysfunctional emotion regulation strategies, even after cessation of NSSI. One recent study [13••] was able to show that adolescents with ceased repetitive NSSI were very likely to show high levels of substance misuse. Furthermore, NSSI is a significant risk factor for suicide attempts [14] and suicides [15]. A recent study reported increased risk of suicide in individuals cutting themselves on other body areas than arms or wrists [16•]. An association of earlier age of onset with an increased risk of developing BPD later on in life was found in a follow-up study in young adults who had engaged in NSSI as adolescents [17].

In summary, NSSI is very present internationally in adolescent populations both in community as well as in clinical samples. Even though NSSI seems to cease in late adolescence or early adulthood in most affected persons, the behavior has high clinical implications due to the various long-term risks involved.

## Etiology and Risk Factors

The following sections describe specific risk factors that have been identified regarding the development of NSSI and are part of its etiology. In a recent meta-analysis on risk factors of NSSI, overall risk factors with the strongest effects (OR > 3.0) included a former history of NSSI, cluster B personality disorders, and hopelessness. Associations for an OR > 2 were found for prior suicidal thought/behaviors, exposure to peer NSSI, patient prediction (self-reported likelihood of engaging in NSSI in the future), and abuse [18]. However, the overall weighted mean ratio was rather low (OR = 1.56 and OR = 1.16 after adjusting for publication bias) and confidence intervals (especially for cluster B personality disorders) were quite high. Furthermore, these results were moderated by NSSI measurement, sample age and type (clinical or general population), and prediction case measurement (binary or continuous). Therefore, results of the meta-analysis have to be interpreted with care and the authors call the need for standardized NSSI measurements in longitudinal studies on NSSI.

### Demographic Factors

As described above, NSSI most commonly occurs in early to mid-adolescence and commonly ceases in young adulthood. Adolescence is a vulnerable phase for developing NSSI, as elevated levels of impulsivity and emotional reactivity are present due to brain developmental processes [19]. Apart from age, female gender has been identified as a risk factor for NSSI. A recent meta-analysis [20•] reported that female adolescents and adults were more likely to engage in NSSI than males. This difference was larger in clinical populations as compared to studies conducted in the general population. Female participants are also more likely to engage in cutting as a method of NSSI, as are males [21•]. In addition, Barrocas and colleagues [22] found cutting to be the most common method for girls and hitting against a wall to be the most common method for boys. Regarding cognitive factors, in a study including 4810 adolescents aged 16 to 17 years, higher IQ was associated with a higher risk of engaging in NSSI [23].

### Social Factors

In a prospective longitudinal study across 2.5 years, Hankin and colleagues [24] were able to show dysfunctional relationships to be a significant risk factors for NSSI. In the same line, bullying has been shown to be a risk factor for the development of NSSI repeatedly. Lereya and colleagues [25] found being bullied by peers in childhood and early adolescence to be a greater risk for self-harm in adulthood than being maltreated by parents in two large longitudinal samples (Avon Longitudinal Study of Parents and Children in the UK (ALSPAC) and Great Smoky Mountains Study in the US (GSMS)). A large European study (*N* = 12,068 adolescents from 11 countries) found bullying to be highly associated with engaging in self-harming behaviors [26]. NSSI being associated to social contagion was found in 16 studies in a systematic review [27]. However, five of the studies conducted in this review had been published more than 20 years ago, with major methodological flaws. Results from this review suggest that particularly the initial engagement in NSSI might be highly influenced by social contagion (i.e., friends or acquaintances engaging in NSSI, or being exposed to NSSI in the media, especially the internet), while the maintenance of NSSI is very likely rather be related to intrapersonal functions (see below) which develop over time. In the line of social influences, identifying with a certain youth subculture (i.e., *gothic* or *emo*) has shown to increase the risk of engaging in NSSI [28, 29]. Another aspect of social influences is the sexuality of a person in association with norms and values the person has internalized or is common in society. A number of studies have shown non-heterosexual orientation to be strongly linked to the risk of engaging in NSSI [30].

### Media Influence

Regarding the worldwide spread of NSSI, internet use and especially the use of social media has been of increasing interest to researchers to understand dissemination of NSSI content. It has been shown that NSSI-related search terms were sought 42 million times per year on Google [31]. The top 100 YouTube videos with an NSSI content were viewed over two million times, with 90% of non-character videos showing NSSI photographs and 28% of character videos showing NSSI action [32]. In an analysis of the “Yahoo! Answers” database, it was shown that most questions related to NSSI (30.6%) were posted with the intent of seeking validation for NSSI [33], thus providing a possible explanation for the reasons of posting NSSI content. This notion is further supported by a recent study, stating that one third of youth (14 to 25 years) with a history of NSSI reported online help-seeking for NSSI [34]. Therefore, the online activity regarding NSSI can be viewed as beneficial (e.g., decreasing social isolation, receiving encouragement for recovery, reducing urges to self-injure) or potentially harmful (e.g., triggering urges to self-injure, social reinforcement of NSSI) [35]. Future research will have to explore which measures need to be taken in order to use the beneficial potential of online resources while restricting harmful consequences.

### Adverse Childhood Events

The risk for engaging in NSSI seems to be elevated by the experience of adverse childhood events like parental neglect, abuse, or deprivation [36, 37]. However, recent research provides more differentiated findings on the experience of adverse childhood events. In a study by Thomassin et al. [38], only child emotional abuse remained significantly associated with NSSI, when different types of adverse childhood experiences were analyzed simultaneously. This is in line with a review and a meta-analysis, finding the experience of sexual abuse to be only moderately linked to the development of NSSI [39, 40]. In another study, only indirect childhood maltreatment (i.e., witnessing domestic violence) was significantly associated with NSSI, and direct forms of maltreatment (physical or sexual abuse) were not. However, emotional abuse was not assessed in this study. Furthermore, a strong association of increased parental critique or parental apathy has been shown repeatedly [8, 41].

### Neurobiological Factors

Research on neurobiological factors regarding the development and continuation of self-injurious behaviors has mostly been conducted in adults with borderline personality disorder [42–44]. However, some studies have focused on neurobiological alterations in adolescents engaging in NSSI. However, since none of those studies are longitudinal studies, results should be interpreted with care and rather represent correlates with NSSI than risk factors. As NSSI is often associated with stressful events or situations (see below), and the hypothalamic-pituitary adrenocortical (HPA) axis is involved in coping with stressful situations [45], studies testing a relationship between NSSI and the HPA axis have been conducted, all showing an altered pattern of HPA axis regulation. A recent study measured salivary and hair cortisol in 26 adolescents with NSSI and compared them to healthy controls. Although no alterations were found with regards to hair cortisol, individuals with NSSI showed higher cortisol awakening responses [46]. In another recent study, the dexamethasone suppression test (DST) was used to evaluate HPA axis response in adolescent girls with depressive symptomatology, half of whom showed a history of NSSI. Individuals with NSSI showed lower post DST cortisol levels, indicative of an efficient negative feedback loop [47]. One study by Kaess et al. [48] found decreased levels of cortisol in adolescents with NSSI in response to the Trier social stress test, which could point towards a hypo-responsiveness of the HPA axis in adolescents with NSSI in acutely stressful situations. Another study focusing on socially stressful situations investigated differences in the neural processing of social exclusion using functional magnetic resonance imaging (fMRI). In this study, Groschwitz et al. [49•] found differentiated processing of social exclusion in depressed adolescents with NSSI as compared to adolescents with mere major depression and healthy controls. Those differences were mainly prevalent in the medial prefrontal cortex (mPFC) and the ventrolateral prefrontal cortex (vlPFC), which might point towards adolescents with NSSI being more affected by social exclusion. These studies add further evidence to the link between interpersonal stressors and NSSI, which was also addressed in one of the few studies searching for genetic factors in NSSI. A gene × environment interaction was shown in two independent adolescent community samples. Adolescents, who carried at least one short allele of the transporter-linked polymorphic region (5-HTTLPR) of the SLC6A4 gene coding for the serotonin transporter and were exposed to severe interpersonal stress, showed an elevated likelihood of engaging in NSSI [50]. In another study applying fMRI in adolescents with NSSI, differentiations in the limbic system as compared to healthy controls were found when processing emotional stimuli [51].

Regarding the perception and processing of physical pain, results from adolescent samples have so far shown rather inconsistent results. One study in adolescents with BPD showed elevated pain thresholds [52]. In another study in youth (aged 16–24) with comparable rates of BPD in both the NSSI and the clinical control group, no differentiation with regard to pain threshold or processing of physical pain was shown [53]. However, differences during pain offset were shown in behavioral data (higher reported feelings of relief), as well as in neuroimaging data (in reward-related areas [53]). Pain offset relief (i.e., the removal of physical pain) has been associated with a simultaneous beneficial effect on emotions [54]. This could point towards pain offset playing an important role in the automatic negative reinforcement of NSSI (see below). On a neurobiological level, differentiations in the processing of physical pain might be related to decreased levels of β-endorphin and Met-enkephalin in the liquor cerebrospinalis, which in turn might be related to deficits in the reaction to external stressors in patients with NSSI [55•].

In summary, adolescent age, female gender, social or medial contact with NSSI, bullying, and adverse childhood experiences like emotional abuse or neglect seem to be major risk factors for the development of NSSI. Findings from neurobiological studies point towards abnormalities in the HPA axis, the endogenous opioid system, as well as the neural processing of emotionally, socially, or physically adverse stimuli.

## Functions of NSSI

The four-factor model [56] is often referred to when describing functionalities of NSSI. This model describes intrapersonal (“automatic”) as well as interpersonal (“social”) processes, which can both be positively and negatively reinforcing the behavior. For example, in the line of automatic negative reinforcement, NSSI serves the function of diminishing negative feelings or thoughts (i.e., anger, tension), while automatic positive reinforcement describes the experience of pleasant or positive feelings or thoughts during or after engaging in NSSI (i.e., feeling alive). Social positive reinforcement describes reinforcing social interaction (i.e., getting attention or sending a message to others), while social negative reinforcement describes NSSI as a means to escape unpleasant social interactions (i.e., ending an argument, not attending sports class).

Most studies have found automatic negative reinforcement to be the most common function when endorsing in NSSI [17, 57, 58]. Studies using economical momentary assessment (EMA) have shown increased feelings of loneliness, sadness, and feeling overwhelmed before engaging in NSSI [59]. In a systematic review, Hamza and Willoughby [60] found the administration of physical pain to be associated with a decrease in negative affect in all studies.

## Therapy for NSSI in Adolescence

### Surgical Treatment

In more severe forms of NSSI, a surgical treatment of wounds can be necessary. In those cases, a good cooperation between different professions is very beneficial (given the consent of the patients and their caregivers). Furthermore, negative emotions towards NSSI or the patient should not be displayed. For example, one study [61] showed that negative reactions of adults towards NSSI of an adolescent can lead to avoidance of help-seeking in the future.

### Psychotherapy

Even though the number of publications on treatment specifically designed for NSSI is rather low, there is steadily growing evidence of the effectiveness of psychotherapeutic treatments for NSSI in adolescents. A recent systematic review mainly identified dialectical behavioral therapy for adolescents (DBT-A), cognitive behavioral therapy (CBT), and mentalization-based treatment for adolescents (MBT-A) to be effective for the therapy of NSSI in adolescence [62•]. In a recent randomized controlled study [63], reduction of NSSI was found to be steady 1 year after DBT-A. The reduction was significantly larger than the reduction of NSSI in a control condition. Significant reduction of NSSI was also shown in a pilot study on DBT-A [64]. A study on a CBT group treatment (“cutting down”) showed significant reduction of NSSI [65]. This program is also currently being evaluated in a larger randomized controlled trial in Germany [66]. MBT-A also showed significant reduction of self-harming behaviors [67] after 12 months of treatment.

In total, no specific treatment showed to be superior to others, or can be recommended as primary option at this stage, as the number of published studies is very small [62•]. The recently developed German clinical guidelines for the treatment of NSSI [68, 69] therefore defined core elements of psychotherapeutic treatment of NSSI in adolescence. Treatments should include the following: establishment of treatment motivation, psychoeducation, identification of factors which trigger or maintain NSSI, conveying alternative behavioral skills to NSSI and conflict resolution strategies, and adherence to guidelines for treating comorbid mental health issues.

### Treatment with Psychiatric Medication

The evidence of psychiatric medication used for the treatment of NSSI in adolescents is still insufficient [70]. Therefore, no such treatment can be recommended. However, sedative medication might be used when a patient is presenting with very high tension in an inpatient setting.

## NSSI as Independent Diagnosis

To date, NSSI is only defined as a symptom of borderline personality disorder (BPD) or as “intentional self-injury with a sharp object” (X78) in the classification system of the World Health Organization (ICD-10). In the DSM-5 [4], NSSI has been suggested as possible independent diagnostic entity (NSSID) in section 3, condition for further study. The repetitive nature of NSSI (at least five times within the last year), as well as its functionalities (i.e., to reduce negative emotional states), is included in this definition. Furthermore, the definition clearly distinguishes NSSI from suicidality, and therefore also from the definition of “deliberate self-harm” (DSH), which includes all self-injurious acts, despite their intention (suicidal or non-suicidal). In recent years, a number of studies have tried to validate NSSID criteria (e.g., [9, 10, 58, 71–73]. However, validation of NSSID is still in progress and has led to an ongoing discussion. Some researchers support the inclusion of NSSID, as NSSI can lead to impairment without comorbid psychiatric disorders [9, 74] and results of several studies suggest that it might occur independently of BPD in a large number of adolescents (e.g., [10]). Defining NSSID could lead to enhanced development and evaluation of specific treatments for NSSI, as well as monetary means to treat NSSI without comorbid psychopathology in health insurance systems of many countries [75, 76]. However, other researchers (e.g., [77, 78]) criticize the defined dichotomy between non-suicidal and suicidal behaviors. An alternative suggestion is to view NSSI and suicide on a continuum of self-harming behaviors. The argument is that with a dichotomous perception, practitioners might underestimate the risks involved with NSSI [78]. Furthermore, they see a risk of stigmatizing a large number of adolescents as “mentally ill,” even though NSSI ceases in late adolescence in most cases. Furthermore, it has been argued that also non-suicidal self-poisonings do exists, which would not be adequately addressed in the recent DSM-5 definition of NSSID [79]. Another criticism of NSSID is that there is still no sufficient evidence of effective treatments, and it could be problematic to define a disorder without specific treatments in place. However, one could argue that there are a number of diseases that are listed in classification systems (e.g., certain types of cancer), for which no effective treatments have yet been developed.

Overall, despite some risks involved with defining NSSI as independent disorder, and criteria still being in need of further validation, this definition could lead to enhancements in communication, research, prevention, and intervention.

## Conclusion

Epidemiological studies have shown that NSSI is a major concern in adolescence. Despite its cessation in late adolescence to early adulthood, the risk for long-term mental health issues, suicidality, and engaging in risk-taking behaviors is elevated in individuals, who engaged in NSSI during their adolescent years repeatedly. While a number of studies support bullying, negative social interactions, and emotional childhood abuse to be risk factors for NSSI, childhood sexual and physical abuse show much weaker evidence. Neurobiological findings point towards alterations in the HPA-axis and the endogenous opioid system, as well as the neural processing of emotional stimuli. Therapy for NSSI has greatly been derived from therapy approaches for BPD. Despite DBT-A, MBT-A, and CBT showing good effectiveness, more studies are necessary to validate those findings. So far, there is no sufficient evidence for pharmacological treatments for NSSI in adolescents.

In summary, while to date there is good international evidence on the prevalence of NSSI in adolescence, as well as the functionalities of NSSI, much research is still needed on the etiology (especially the neurobiological aspects) and treatment of NSSI in adolescence, including possible short-term interventions for less severe NSSI symptomatology. The ongoing debate of including NSSI as independent disorder in the DSM-5 has sparked new research and could significantly improve knowledge about the etiology, phenomenology, and treatment of NSSI.
